# Supplementary material for: A requirement of Polo-like kinase 1 in murine embryonic myogenesis and adult muscle regeneration
Source: eLife. 2019 Aug 8;8:e47097. doi: 10.7554/eLife.47097 (PMC6687435; doi:10.7554/eLife.47097)
Supplement: Supplementary file 1. [file elife-47097-supp1.pdf]

Supplementary File 1. Genotypes and distribution of *Plk1* conditional knockout embryos

GENOTYPES	$MYOD^{CRE}PLK^{F/-}$	$MYOD^{CRE}PLK^{F/F}$	$PLK^{F/-}$	$PLK^{F/F}$	*TOTAL*
**ACTUAL NUMBERS**	18	13	17	21	69
**PREDICTED NUMBERS**	17.25	17.25	17.25	17.25	69

Chi-square test: $p = 0.596462458$
