# Supplementary material for: A requirement of Polo-like kinase 1 in murine embryonic myogenesis and adult muscle regeneration
Source: eLife. 2019 Aug 8;8:e47097. doi: 10.7554/eLife.47097 (PMC6687435; doi:10.7554/eLife.47097)
Supplement: Supplementary file 2. [file elife-47097-supp2.docx]

**Supplementary File 2.** Primers used in this study

| Primer | Sequence(5’—3’) |
| --- | --- |

| *Plk1*  *Plk2*  *Plk3*  *Plk4*  *MyoG*  *Pax7*  *eMyhc*  *Plk1* genotyping  *Plk1* recombination | F: TAATGACTCAACACGCCTGATT  R: AGCTCAGCAGCTTGTCTACCAT  F: CCTGCGGACTATCACCTACCA  R: CTGCCCATCTTCAGAAGGCT  F: GCACATCCATCGGTCATCCAG  R: GCCACAGTCAAACCTTCTTCAA  F: AGGAGAAACTAATGAGCACCACA  R: TGGCTCTCGTGTCAGTCCAA  F: TGCCCAGTGAATGCAACTCC  R: TTGGGCATGGTTTCGTCTGG  F: TCTCCAAGATTCTGTGCCGAT  R: CGGGGTTCTCTCTCTTATACTCC  F: AAAAGGCCATCACTGACGC  R: CAGCTCTCTGATCCGTGTCTC  F: ACAGCGACTTTGTATTTGTAGTTTTG  R: CACTTTATGAATCCATTTCCTGTACC  R: TTTCAGCTTAGTAAAGAGACA |
| --- | --- |
