# Supplementary material for: A requirement of Polo-like kinase 1 in murine embryonic myogenesis and adult muscle regeneration
Source: eLife. 2019 Aug 8;8:e47097. doi: 10.7554/eLife.47097 (PMC6687435; doi:10.7554/eLife.47097)
Supplement: Transparent reporting form [file elife-47097-transrepform.docx]

***eLife’s* transparent reporting form**

We encourage authors to provide detailed information *within their submission* to facilitate the interpretation and replication of experiments. Authors can upload supporting documentation to indicate the use of appropriate reporting guidelines for health-related research (see [EQUATOR Network](http://www.equator-network.org/%20)), life science research (see the [BioSharing Information Resource](https://biosharing.org/)), or the [ARRIVE guidelines](http://www.plosbiology.org/article/info:doi/10.1371/journal.pbio.1000412) for reporting work involving animal research. Where applicable, authors should refer to any relevant reporting standards documents in this form.

If you have any questions, please consult our Journal Policies and/or contact us: [editorial@elifesciences.org](mailto:editorial@elifesciences.org).

**Sample-size estimation**

- You should state whether an appropriate sample size was computed when the study was being designed
- You should state the statistical method of sample size computation and any required assumptions
- If no explicit power analysis was used, you should describe how you decided what sample (replicate) size (number) to use

Please outline where this information can be found within the submission (e.g., sections or figure legends), or explain why this information doesn’t apply to your submission:

This study mainly focuses on analyses of mice (wildtype and knockout) and muscle stem cells isolated from the mice that do not involve large sample size. Although no explicit power analysis was performed to compute sample size, the sample size was determined based on previous empirical and power analyses used in similar studies. Sample size (n, biological replicates) for each experiment is indicated in the figure legends.

**Replicates**

- You should report how often each experiment was performed
- You should include a definition of biological versus technical replication
- The data obtained should be provided and sufficient information should be provided to indicate the number of independent biological and/or technical replicates
- If you encountered any outliers, you should describe how these were handled
- Criteria for exclusion/inclusion of data should be clearly stated
- High-throughput sequence data should be uploaded before submission, with a private link for reviewers provided (these are available from both GEO and ArrayExpress)

Please outline where this information can be found within the submission (e.g., sections or figure legends), or explain why this information doesn’t apply to your submission:

Sample size (n) for each experiment is indicated in the figure legends, all n numbers represent biological replicates. Two to three technical replicates are typically performed and averaged within each biological sample. Specifically, quantitative real-time PCR analysis was performed in biological triplicates (Fig1D, 1E, 1F) and quadruplets (Fig1A, 1B, 2C). Plk1 Immunofluorescence was perform in technical (Fig 2A) and biological (Fig S2B) triplicates. All other Immunofluorescence assay was perform in both biological replicates and technical triplicates (Fig4G, 6E, 6F, 7C, 7E, 7H, S2B, S2E, S3F, S3H, S4A, S4D, S5C ) by either examining three sections in the same muscle or repeated three times with the same cells. Detailed counting method was provided in *Methods*: *Isolation and culture of single fiber* and *Hematoxylin–eosin and immunofluorescence staining*. PI staining (Fig 5F) and cell number counting (Fig 5E, S4B, S5A, S5B) was performed in both biological and technical triplicates. For cell analysis, each technical replicate involves 25-50 cells. The technical replicates were averaged to obtain each biological replicate value. Injured and non-injured TA weight were obtained from 4 individual mice for biological quadruplets.

No outliers were excluded from the data.

**Statistical reporting**

- Statistical analysis methods should be described and justified
- Raw data should be presented in figures whenever informative to do so (typically when N per group is less than 10)
- For each experiment, you should identify the statistical tests used, exact values of N, definitions of center, methods of multiple test correction, and dispersion and precision measures (e.g., mean, median, SD, SEM, confidence intervals; and, for the major substantive results, a measure of effect size (e.g., Pearson's r, Cohen's d)
- Report exact p-values wherever possible alongside the summary statistics and 95% confidence intervals. These should be reported for all key questions and not only when the p-value is less than 0.05.

Please outline where this information can be found within the submission (e.g., sections or figure legends), or explain why this information doesn’t apply to your submission:

Unless otherwise stated, statistical analyses were performed using two-tailed student’s T-test and all data were presented as mean ± SEM. P values <0.05 or <0.01 were considered statistically significant and were all shown in the figure legends. Chi-square test was used in Table S3 for analysing Mendelian inheritance.

(For large datasets, or papers with a very large number of statistical tests, you may upload a single table file with tests, Ns, etc., with reference to sections in the manuscript.)

**Group allocation**

- Indicate how samples were allocated into experimental groups (in the case of clinical studies, please specify allocation to treatment method); if randomization was used, please also state if restricted randomization was applied
- Indicate if masking was used during group allocation, data collection and/or data analysis

Please outline where this information can be found within the submission (e.g., sections or figure legends), or explain why this information doesn’t apply to your submission:

Samples (tissues and cells) were allocated based on the genotyping of the mice. Siblings were used for comparison between wildtype and mutant mice. In pharmacological treatments, vehicle (solvent) control were used. Specifically, BI or Vehicle treatment as shown in *Methods*: *Mice and animal care* and *Isolation, culture and differentiation of primary myoblast*. *Plk1^PKO^* (*Pax7^CreER^*; *Plk1^f/f^*) and WT (*Plk1^f/f^*) were used in Fig 4, 5B, S1 and S2. *Plk1^MKO^* (*MyoD^Cre^*; *Plk1^f/f^*) and WT (*Plk1^f/f^*) were used in Fig 2. *Plk1-null* (4OHT induced *Plk1* KO) and WT (*Plk1^f/f^*) myoblast were used in Fig 5D, 5E, 5F, 5G 6, 7A, 7B, 7C, 7F, 7G, 7H and S4B. BI2536 and Vehicle treatment were used in Fig 7D, 7E, S3, S4C, S4D, S5, S6C and S6D. Samples were randomized but comparisons were made between animals of identical age and sex in this study.

**Additional data files (“source data”)**

- We encourage you to upload relevant additional data files, such as numerical data that are represented as a graph in a figure, or as a summary table
- Where provided, these should be in the most useful format, and they can be uploaded as “Source data” files linked to a main figure or table
- Include model definition files including the full list of parameters used
- Include code used for data analysis (e.g., R, MatLab)
- Avoid stating that data files are “available upon request”

Please indicate the figures or tables for which source data files have been provided:

No additional data files were provided but we will be happy to supply all original data upon acceptance of the manuscript.
